# The Scandal of Out-Patients: A Suggestion

**Published:** 1911-10-14

**Authors:** James Ashenden


*To the Editor of* The Hospital.

### THE SCANDAL OF OUT-PATIENTS: A SUGGESTION.

22 Albemarle Gardens, New Malden,

October 9, 1911.

*To the Editor of* The Hospital.

Sir,—I have read with interest the comments of Dr. Stephen Andrew on the out-patient departments of hospitals, and would suggest the following as a remedy remarking the hygienic aspect of same: To have a series of waiting-rooms on either side of the corridor, several set apart for patients suffering from disease, and the others for children and accidents. The rooms set apart for diseases to have a ventilator in the ceiling communicating directly with the external air (*i.e.* the waiting-rooms to be on one floor only), and arranged in size so that each patient has from 1,000 to 1,500 cubic feet of air space, and generally warmed up to 60° F. or more, according to the nature of the disease. The windows should be fitted with a draught-proof device whereby fresh air may be obtained without draught. I may say that I have constructed such a device, with scarcely any extra expense.

I think these few suggestions will to a certain extent rectify the uncomfortable and evil-smelling element as far as hygiene is concerned. I will send you a drawing explaining the above should you wish it; also of the draught-preventing device. Trusting I have not wearied you with the foregoing, which is written with desire to help in the matter.—I remain, dear Sir, yours faithfully,

James Ashenden.

[We shall be very glad to see and publish Mr. Ashenden's plans.—Ed., The Hospital.]

